# Supplementary material for: Dirac-fermion-assisted interfacial superconductivity in epitaxial topological-insulator/iron-chalcogenide heterostructures
Source: Nat Commun. 2023 Nov 6;14:7119. doi: 10.1038/s41467-023-42902-2 (PMC10628154; doi:10.1038/s41467-023-42902-2)
Supplement: Supplementary file 1 — Supplementary Information [file 41467_2023_42902_MOESM1_ESM.pdf]

Supplementary Information

Dirac-Fermion-Assisted Interfacial Superconductivity in Epitaxial Topological-Insulator/Iron-Chalcogenide Heterostructures

Hemian Yi[1,#], Lun-Hui Hu[1,2,#], Yi-Fan Zhao[1], Ling-Jie Zhou[1], Zi-Jie Yan[1], Ruoxi Zhang[1], Wei Yuan[1], Zihao Wang[1], Ke Wang[3], Danielle Reifsnyder Hickey[3,4,5], Anthony R. Richardella[1], John Singleton[6], Laurel E. Winter[6], Xianxin Wu[7], Moses H. W. Chan[1], Nitin Samarth[1], Chao-Xing Liu[1*], and Cui-Zu Chang[1,3*]

[1]Department of Physics, The Pennsylvania State University, University Park, PA 16802, USA

[2]Department of Physics and Astronomy, The University of Tennessee, Knoxville, TN 37996, USA

[3]Materials Research Institute, The Pennsylvania State University, University Park, PA 16802, USA

[4]Department of Chemistry, The Pennsylvania State University, University Park, PA 16802, USA

[5]Department of Materials Science and Engineering, The Pennsylvania State University, University Park, PA 16802, USA

[6]National High Magnetic Field Laboratory, Los Alamos, NM 87544, USA

[7]CAS Key Laboratory of Theoretical Physics, Institute of Theoretical Physics, Chinese Academy of Sciences, Beijing 100190, China

[#] These authors contributed equally.

Corresponding authors: cxl56@psu.edu (C.-X. L.); cxc955@psu.edu (C.-Z. C.)

Contents:

I. Supplementary Figures

II. Supplementary Text

1. Influence of disorder effect on interfacial superconductivity in TI/FeTe

2. Massless and massive Dirac fermions in TI

3. Hall traces of the $(Bi_{1-x}Sb_x)_2Te_3$/FeTe heterostructures

References

I. Supplementary Figures

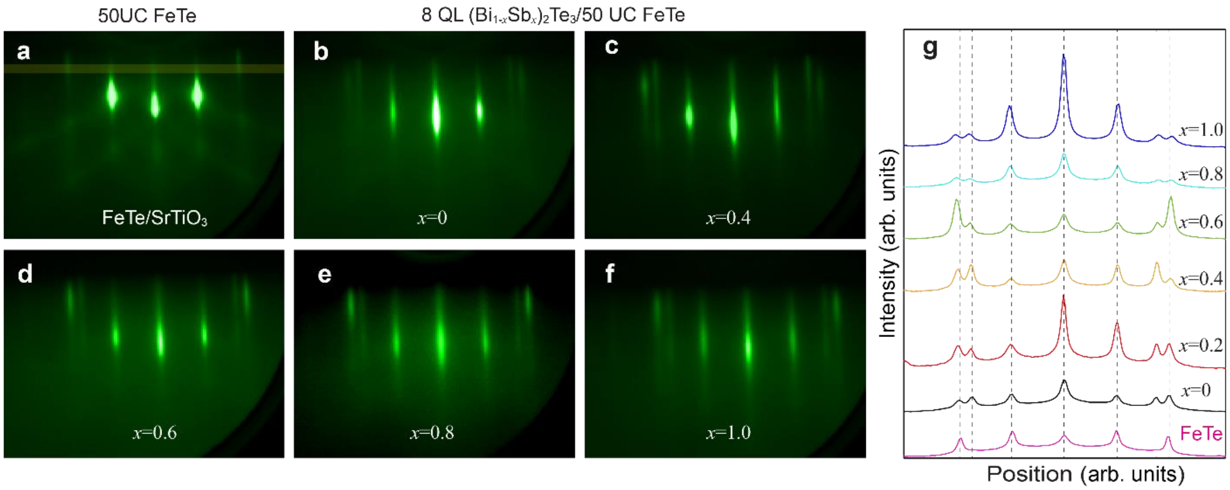

Supplementary Fig. 1| RHEED patterns of $(Bi_{1-x}Sb_x)_2Te_3$/FeTe heterostructures. a, 50 UC FeTe on a heat-treated $SrTiO_3(100)$ substrate. **b-f**, 8 QL $(Bi_{1-x}Sb_x)_2Te_3$/50 UC FeTe heterostructures with x=0 (b), x=0.4 (c), x=0.6 (d), x=0.8(e), x=1(f). **g**, RHEED pattern intensity of the narrow streaks as displayed in (a). The dashed lines indicate the peak position for the x=0 heterostructure.

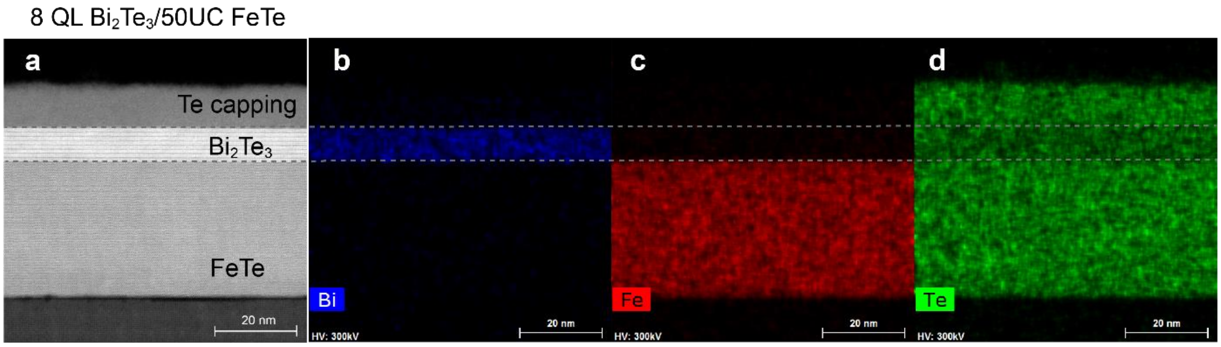

Supplementary Fig. 2| ADF-STEM image and corresponding EDS maps of the Bi_2Te_3/FeTe heterostructure. a, The ADF-STEM image of the 8 QL Bi_2Te_3/50 UC FeTe heterostructure. **b-d**, The corresponding EDS maps of Bi, Fe, and Te of the 8QL Bi_2Te_3/50UC FeTe heterostructure.

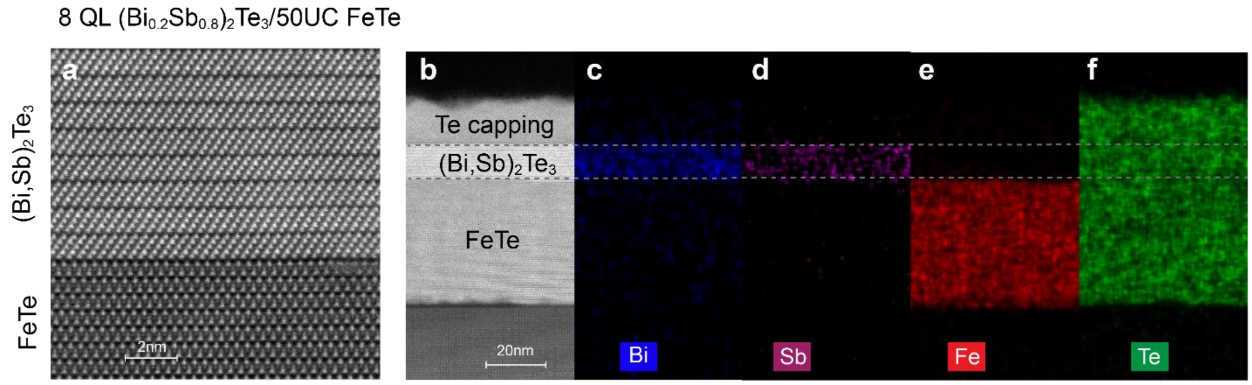

Supplementary Fig. 3| ADF-STEM image and corresponding EDS maps of the $(Bi_{0.2}Sb_{0.8})_2Te_3$/FeTe heterostructure. a,b, The ADF-STEM images of the 8 QL $(Bi_{0.2}Sb_{0.8})_2Te_3$/50 UC FeTe heterostructure. **c-f**, The corresponding EDS maps of Bi, Sb, Fe, and Te of the 8QL $(Bi_{0.2}Sb_{0.8})_2Te_3$/50UC FeTe heterostructure. The Sb $L_{\alpha 1}$ peak and the Te $L_{\alpha 1}$ peak are used to quantify the Sb distribution in (d) and the Te contribution in (f), respectively.

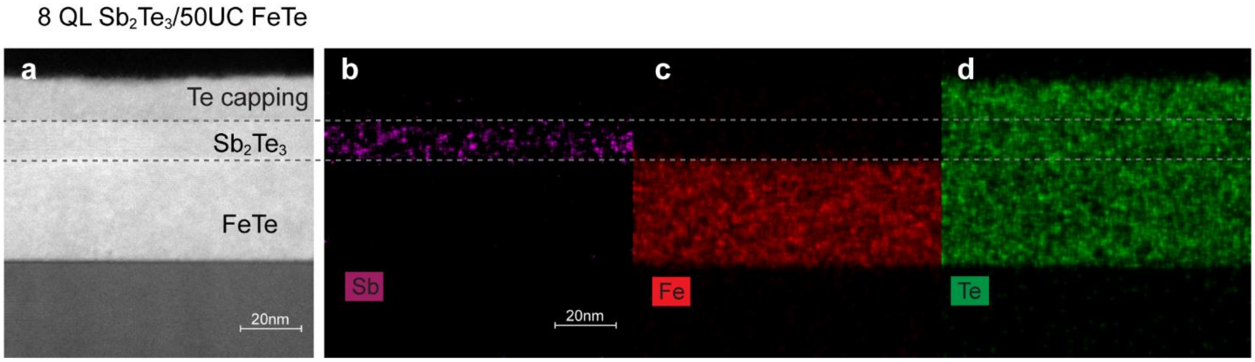

Supplementary Fig. 4| ADF-STEM image and EDS maps of the Sb_2Te_3/FeTe heterostructure. **a**, The ADF-STEM image of the 8QL Sb_2Te_3/50UC FeTe heterostructure. **b-d**, The corresponding EDS maps of Sb, Fe, and Te of the 8QL Sb_2Te_3/50UC FeTe heterostructure. The Sb $L_{\alpha 1}$ peak and the Te $L_{\alpha 1}$ peak are used to quantify the Sb distribution in (b) and the Te contribution in (d), respectively.

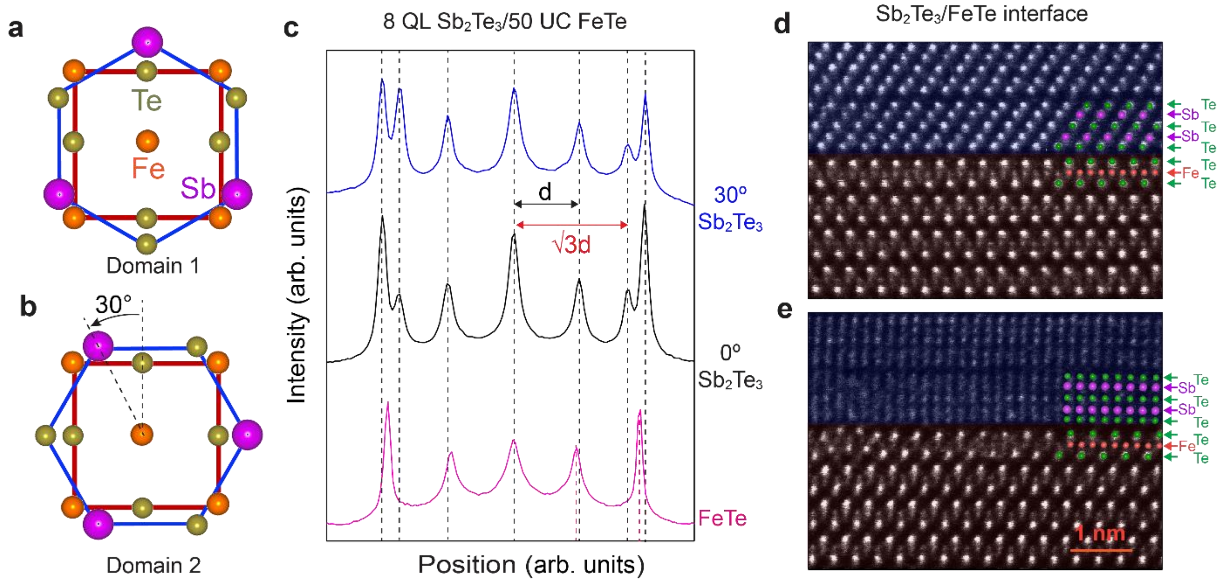

Supplementary Fig. 5| Two domains in Sb_2Te_3/FeTe heterostructures. **a,b,** Schematics of two possible domains. The relative rotation angle is ~30°. **c,** RHEED pattern intensity of the narrow streaks for FeTe and Sb_2Te_3/FeTe heterostructures with two high symmetric angles (i.e. 0° and 30°), respectively. **d,e,** ADF-SSTEM images of Sb_2Te_3/FeTe heterostructure at two different orientations.

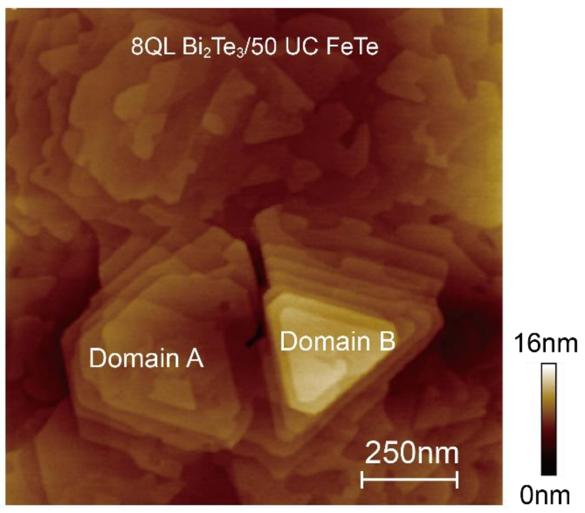

Supplementary Fig. 6| Atomic force microscopy image of the 8 QL Bi_2Te_3/50 UC FeTe heterostructure. The triangular pyramidal structures indicate two different domain orientations.

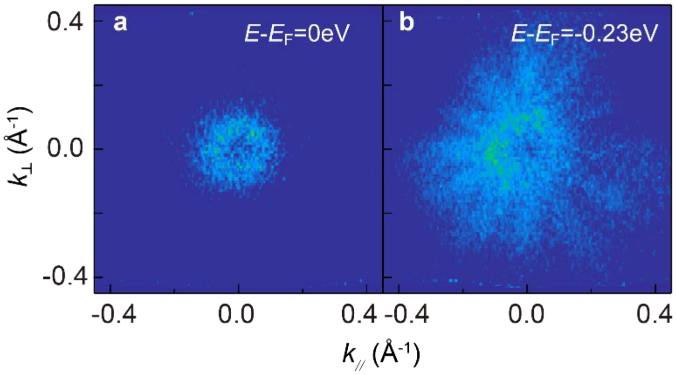

Supplementary Fig. 7| Constant energy contours of the 8 QL Bi_2Te_3/50 UC FeTe heterostructure. a, E-E_F= 0 eV (i.e. Fermi surface). **b**, E-E_F=-0.23eV. The ARPES spectra in (**b**) are composed of both Dirac surface states and bulk valence bands. The appearance of twelve-fold symmetry in the bulk states indicates the existence of the twin domains in the 8 QL Bi_2Te_3 layer.

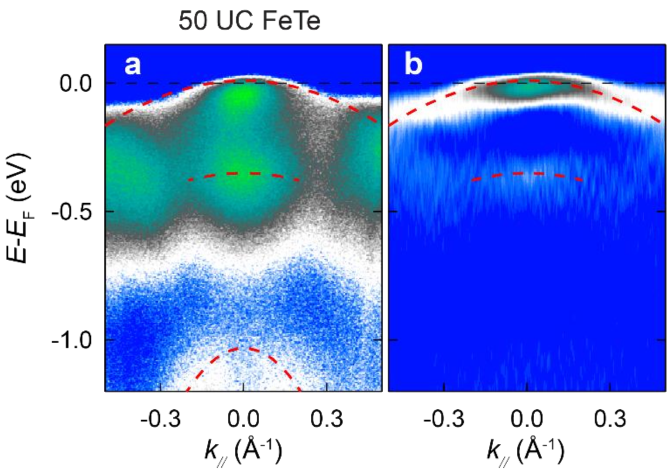

Supplementary Fig. 8| ARPES band map of the FeTe film. a,b, ARPES band map of a 50UC FeTe film (a) and its corresponding second derivative image (b).

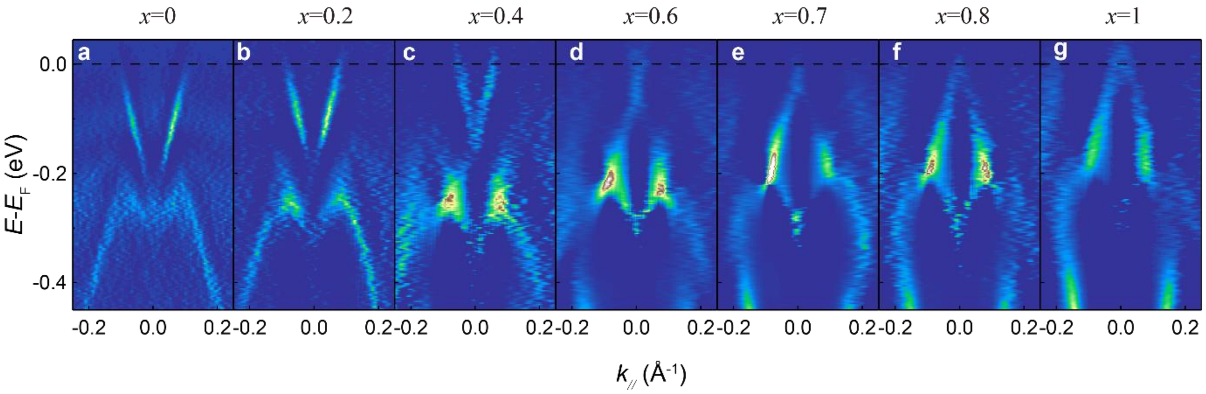

Supplementary Fig. 9| Dirac surface states in 8 QL $(Bi_{1-x}Sb_x)_2Te_3$/50 UC FeTe heterostructures with different Sb concentrations *x*. a-g, Second derivative plots of the ARPES band map in Fig. 2 of the main text.

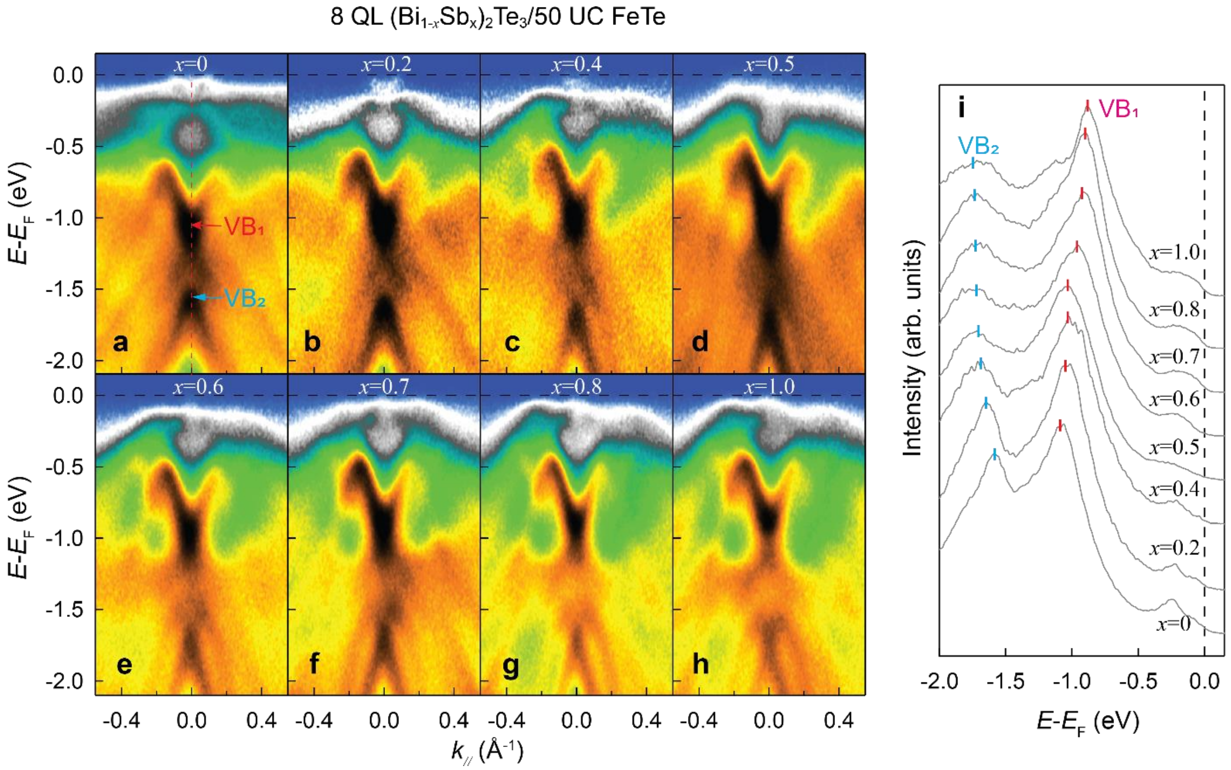

Supplementary Fig. 10| ARPES band maps of $(Bi_{1-x}Sb_x)_2Te_3$/FeTe heterostructures over a wide energy range. a-h, ARPES band maps of 8QL $(Bi_{1-x}Sb_x)_2Te_3$/50UC FeTe heterostructures with x=0 (a), x=0.2 (b), x=0.4 (c), x=0.5 (d), x=0.6 (e), x=0.7(f), x=0.8(g), x=1.0 (h). The colored arrows show two bulk valence bands (VB_1 and VB_2). **i,** The energy distribution curves (EDCs) of 8QL $(Bi_{1-x}Sb_x)_2Te_3$/50UC FeTe heterostructures at the Γ point from ARPES band maps in (a-h).

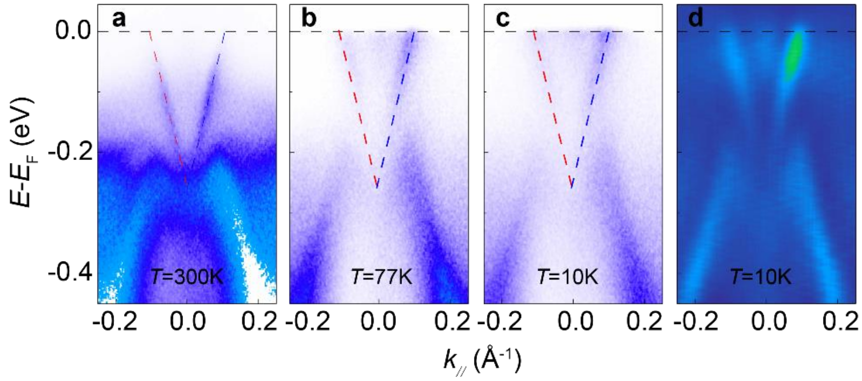

Supplementary Fig. 11| Dirac surface states in the 8 QL Bi_2Te_3/50 UC FeTe heterostructures at different temperatures. a-c, ARPES band maps of 8 QL Bi_2Te_3/50 UC FeTe sample measured at room temperature (*T* ~300 K, **a**), liquid nitrogen temperature (*T* ~77 K, **b**), and liquid helium temperature (*T* ~10 K, **c**). The red and blue dashed lines indicate the Dirac surface states. **d**, Second derivative plot of ARPES band map in (**c**).

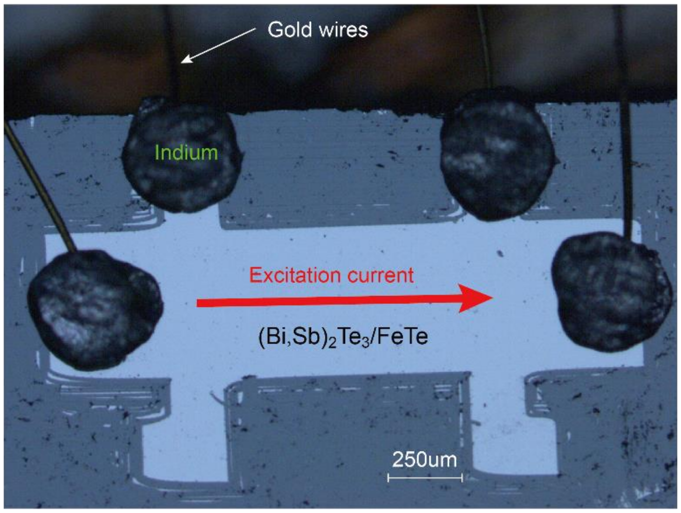

Supplementary Fig. 12| Photograph of the $(Bi,Sb)_2Te_3/FeTe$ Hall bar device used in our transport measurements.

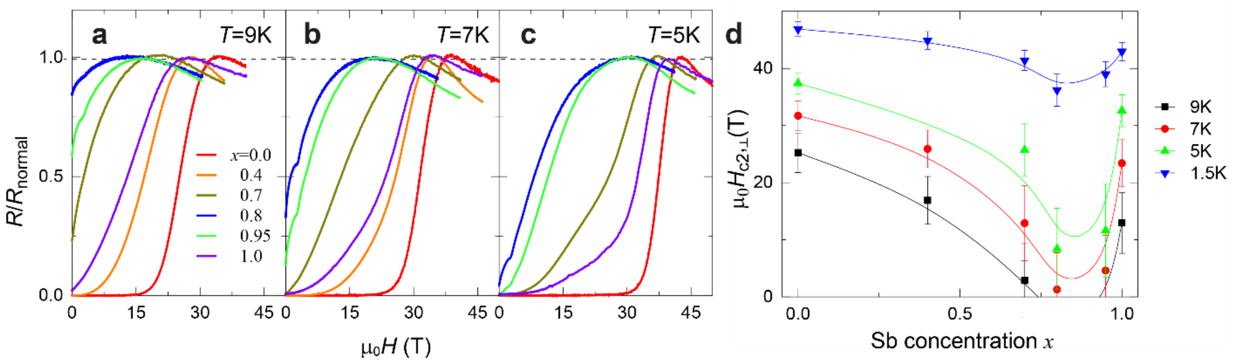

Supplementary Fig. 13| Perpendicular upper critical fields of the $(Bi_{1-x}Sb_x)_2Te_3$/FeTe heterostructures at different temperatures. a-c, $\mu_0 H$ dependence of normalized magnetoresistance R/R_{normal} of 8QL $(Bi_{1-x}Sb_x)_2Te_3$/50UC FeTe heterostructures measured at T =9 K (a), T = 7 K(b), and T =5 K (c). R_{normal} is the normal state resistance of the sample. **d,** The Sb concentration x dependence of the upper critical magnetic field ($\mu_0 H_{c2,\perp}$) at different temperatures. The error bars in (**d**) are estimated to be ~40% of [$\mu_0 H_{c2,\perp}(R_{normal})$- $\mu_0 H_{c2,\perp}(R$~$0.5R_{normal})$].

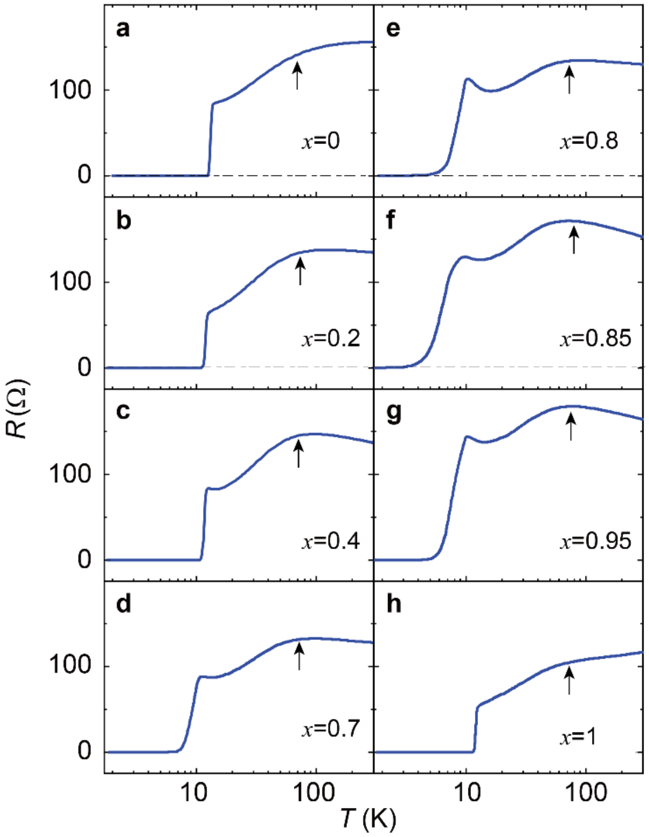

Supplementary Fig. 14| Temperature dependence of the sheet longitudinal resistance *R* of 8 QL $(Bi_{1-x}Sb_x)_2Te_3$/50 UC FeTe heterostructures. a-h, *x*=0 (**a**), *x*=0.2 (**b**), *x*=0.4 (**c**), *x*=0.7 (**d**), *x*=0.8(**e**), *x*=0.85(**f**), *x*=0.95(**g**), and *x*=1(**h**). The arrows indicate the hump features in *R*-*T* curves of these 8 QL $(Bi_{1-x}Sb_x)_2Te_3$/50 UC FeTe heterostructures.

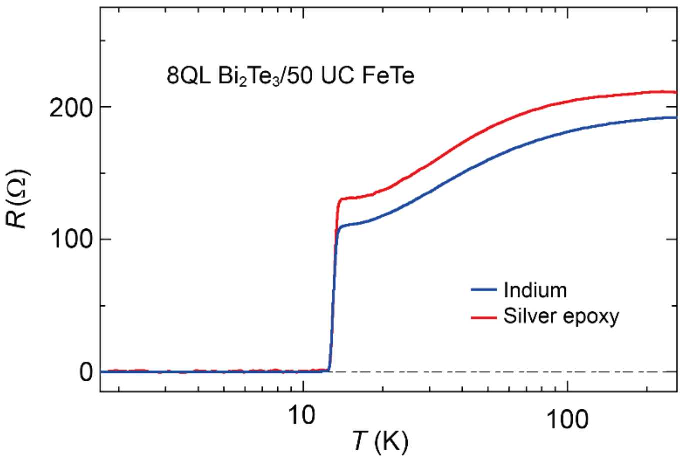

Supplementary Fig. 15| Temperature dependence of the sheet longitudinal resistance *R* of 8 QL Bi_2Te_3/50 UC FeTe measured with indium and silver epoxy contacts. The electrical contacts of the Hall bar devices are made of pressed indium dots (blue) and silver epoxy (red).

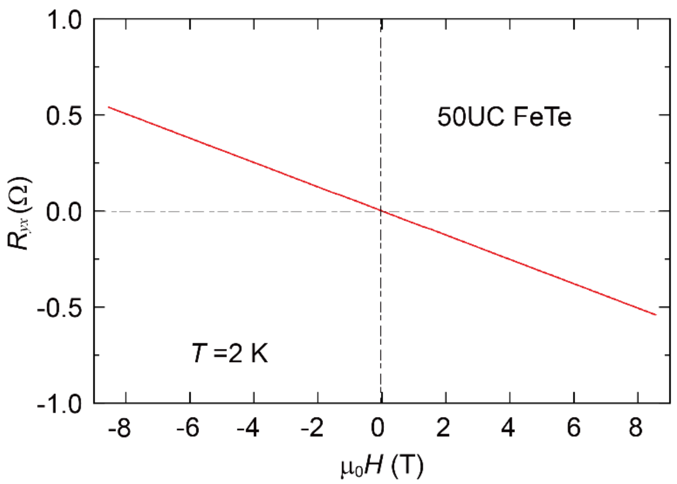

Supplementary Fig. 16| Hall traces of 50 UC FeTe/$SrTiO_3$(100) at *T* =2 K.

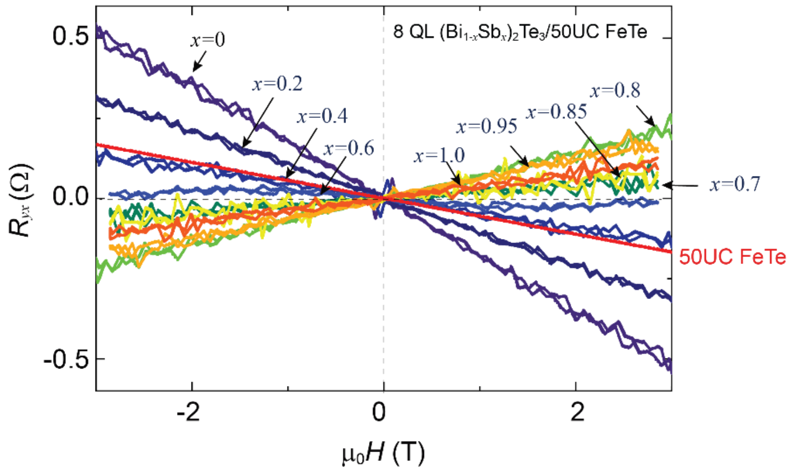

Supplementary Fig. 17| Hall traces of all 8 QL $(Bi_{1-x}Sb_x)_2Te_3$/50UC FeTe heterostructures with interfacial superconductivity and 50 UC FeTe layer without superconductivity. All measurements are taken at T =30 K.

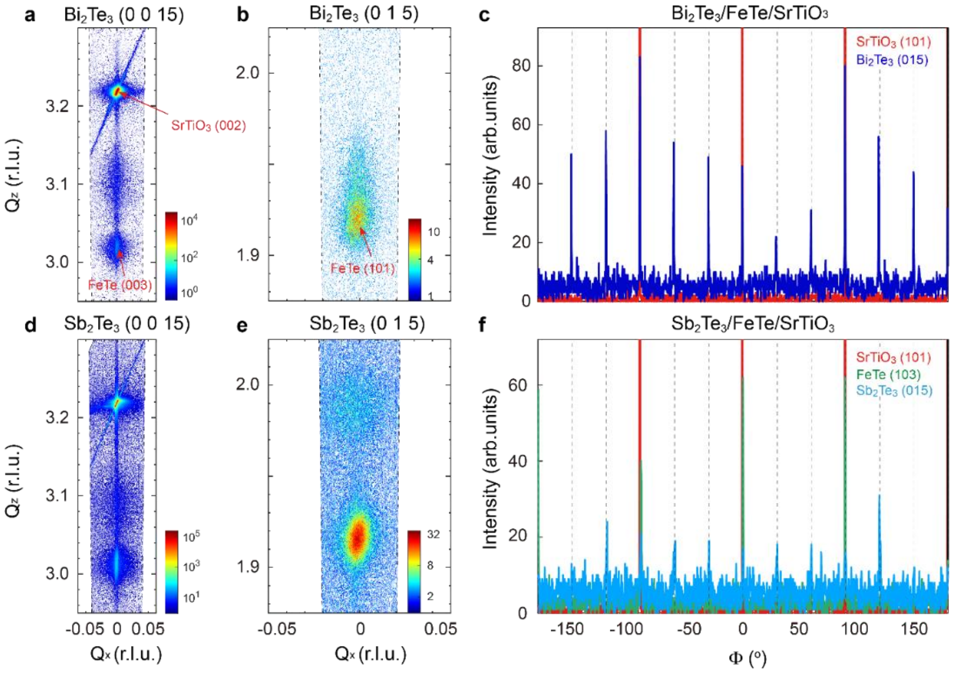

Supplementary Fig. 18| XRD reciprocal space mapping (RSM) of the Bi_2Te_3/FeTe and Sb_2Te_3/FeTe heterostructures. **a**, Symmetric RSM around the Bi_2Te_3 (0 0 15) peak showing $SrTiO_3$ (0 0 2) and FeTe (0 0 3) reflections. The dotted lines show the extent of the scanned region in reciprocal space. **b**, Symmetric RSM measured at χ=58.08° showing Bi_2Te_3 (0 1 5) peak partially overlapped with FeTe (1 0 1). **c**, ϕ scans of Bi_2Te_3 and $SrTiO_3$. Bi_2Te_3 shows twin domains with a 30° rotation. **d**, RSM of Sb_2Te_3 (0 0 15) showing $SrTiO_3$ and FeTe as in (**a**). **e**, RSM measured at χ=58.78° showing Sb_2Te_3 (0 1 5) at a larger Q_Z than the corresponding Bi_2Te_3 peak due to the smaller in-plane lattice constant of Sb_2Te_3. **f,** ϕ scans of Sb_2Te_3, FeTe and $SrTiO_3$. FeTe is epitaxial to $SrTiO_3$ substrate while Sb_2Te_3 shows twin domains with a 30° rotation.

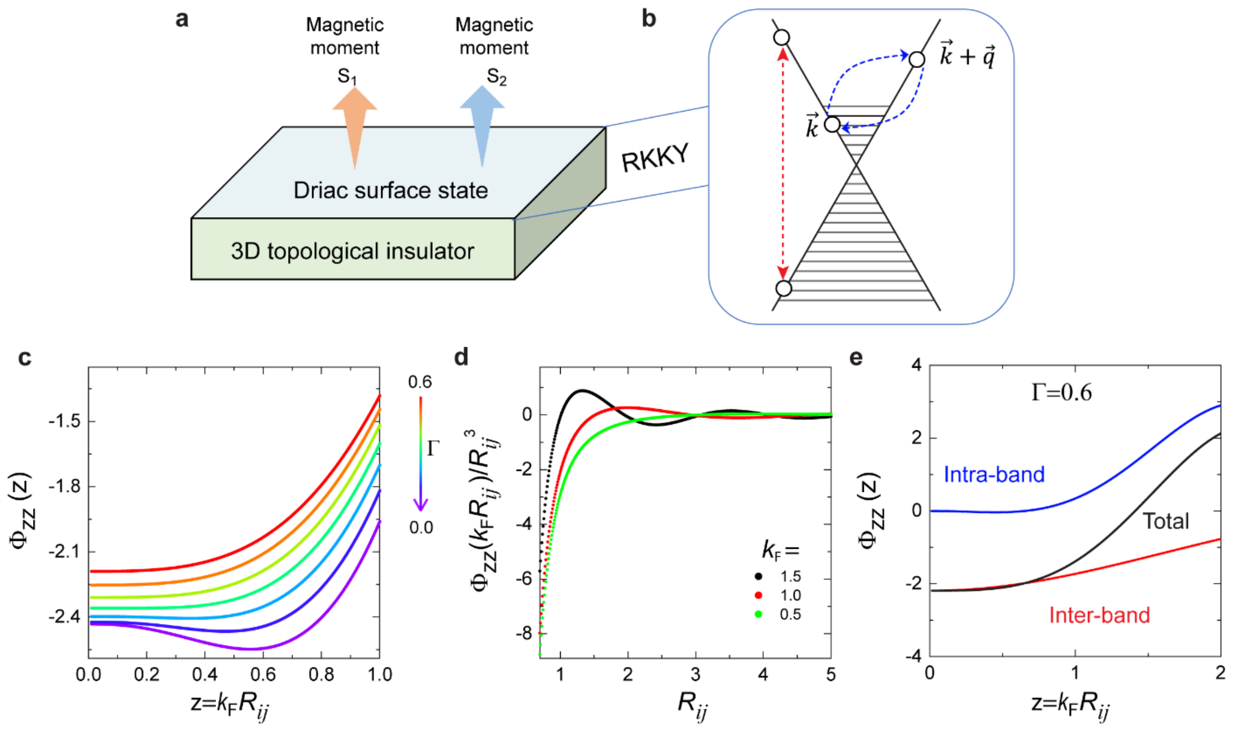

Supplementary Fig. 19| Dirac-electrons-induced RKKY interaction between two adjacent magnetic moments. a, Schematics of two magnetic moments on a TI surface. $\vec{S}_1$ and $\vec{S}_2$ are coupled with each other through exchange spin-spin interaction via itinerant Dirac electrons. **b,** RKKY interaction mechanism between $\vec{S}_1$ and $\vec{S}_2$. The stripe region displays the occupied states of Dirac electrons. The blue and red dashed lines are the intra-band and inter-band scatterings via the exchange coupling between the magnetic moments and Dirac electrons, respectively. The RKKY coupling strength was calculated using the second-order perturbation theory. **c,** RKKY interaction $\Phi_{zz}(z)$ as a function of z at different disorder broadening Γ. $z = k_F R_{ij}$ is a dimensionless parameter, and R_{ij} is the spatial distance between two magnetic moments. For $\Gamma \geq 0.3$, Φ_{zz} shows a monotonic z dependence. **d,** The RKKY interaction strength as a function of R_{ij} at different k_F for a fixed $\Gamma = 0.6$. **e,** RKKY interaction $\Phi_{zz}(z)$ as a function of z at disorder broadening $\Gamma = 0.6$. Both intra-band and inter-band scatterings contribute to the total RKKY interaction. As k_F approaches 0, the contribution from intra-band scattering vanishes.

II. Supplementary Text

1. Influence of the disorder effect on interfacial superconductivity in TI/FeTe

In our theoretical calculations, the influence of the disorder on magnetic interaction can be included in an imaginary self-energy broadening $i\Gamma$ in the Green's function (Methods). The disorder may also give rise to a real part of the self-energy, which might cause a renormalization effect on the Dirac surface states of TI (Ref. [1]). The real part of self-energy can provide corrections to the energy of Dirac points and the velocity of the Dirac surface states, both of which are directly extracted from the experimental data. Therefore, the parameters used in our theory can be treated as "renormalized" values.

Moreover, the disorder in $(Bi_{1-x}Sb_x)_2Te_3$ has been found to be maximized near x=0.5, i.e., the compositions of Bi and Sb are equal, through thermal conductivity measurements [2]. However, in our experiments, the dip feature in both T_c and $\mu_0 H_{c2,\perp}$ appears near x=0.85 (Figs. 4c and 4d), which is far from the position for the maximum disorder but close to the Dirac point of the surface states (i.e., x=0.8) (Fig. 4a). These observations also rule out the possibility that the appearance of the dip features in both T_c and $\mu_0 H_{c2,\perp}$ is a direct result of the disorder effect in the TI layer.

2. Massive and massless Dirac fermions in TI thin films

For the TI thin films in the 2D regime, a hybridization gap is formed between the top and bottom surface states [3-6], which leads to the Dirac fermions acquiring mass (or a gap) and thus makes Dirac fermions massive [7,8]. We note that the terminology of "Dirac fermions" remains consistent regardless of the TI thickness. Our theoretical analysis remains applicable to both massless and massive Dirac fermions. In Supplementary Fig. 19, we have demonstrated that both intra-band scattering and inter-band scattering give rise to the RKKY interaction. In particular, when the chemical potential is close to the Dirac point, the intra-band contribution is negligible due to the vanishing density of states at the Dirac point while the inter-band contribution is the dominant mechanism. In the case of massive Dirac fermions, the inter-band contribution plays a primary role in RKKY interaction and is of ferromagnetic type when the Fermi energy is inside the Dirac fermion gap. This inter-band contribution is also known as the van Vleck mechanism [9] or the Bloembergen-Rowland mechanism [10], which is known to play an important role in inducing ferromagnetism in magnetically doped TI films [11].

3. Hall traces of the $(Bi_{1-x}Sb_x)_2Te_3$/FeTe heterostructures

We perform the Hall measurements on all 8 QL $(Bi_{1-x}Sb_x)_2Te_3$/50UC FeTe heterostructures with interfacial superconductivity (Supplementary Fig. S17) and the 50 UC FeTe layer without superconductivity (Supplementary Figs. S16 and S17). We find that the absolute values of the Hall trace slopes are small for all these samples. Moreover, for $0 \leqslant x \leqslant 0.6$, the Hall traces of these $(Bi_{1-x}Sb_x)_2Te_3$/FeTe heterostructures show systematic behavior, and the Hall trace slope decreases with increasing *x*. However, for *x* is near 0.85, i.e., $0.7 \leqslant x \leqslant 1.0$ and the chemical potential is near the Dirac point of the TI layer, the Hall traces show some fluctuations (Supplementary Fig. S17).

Next, we employ a two-layer structure to model the TI/FeTe heterostructure and performed simple calculations to understand our observations. To simplify our calculations, we ignore the charge transfer effect between TI and FeTe layers. The total Hall resistance of the TI/FeTe bilayer can be calculated using the following equation:

$$R_{yx} = \left(\frac{\frac{R_{yx}{}^{FeTe}}{(R_{xx}{}^{FeTe})^2} + \frac{R_{yx}{}^{TI}}{(R_{xx}{}^{TI})^2}}{\left(\frac{1}{R_{xx}{}^{FeTe}} + \frac{1}{R_{xx}{}^{TI}}\right)^2} \right) \quad (1)$$

Since the FeTe layer exhibits higher conductivity compared to the $(Bi_{1-x}Sb_x)_2Te_3$ layer (Fig. 1c), $R_{xx}{}^{FeTe}$ is much less than $R_{xx}{}^{TI}$ (i.e., $R_{xx}{}^{FeTe} \ll R_{xx}{}^{TI}$) (Ref. [12]). Given this assumption, we can simplify Equation (1):

$$R_{yx} = \left(R_{yx}{}^{FeTe} + R_{yx}{}^{TI} * \left(\frac{R_{xx}{}^{FeTe}}{R_{xx}{}^{TI}}\right)^2 \right) = \left(R_{yx}{}^{FeTe} + \frac{R_{yx}{}^{TI}}{\left(R_{xx}{}^{TI}\right)^2} * \left(R_{xx}{}^{FeTe}\right)^2 \right) \quad (2)$$

Therefore, the total Hall resistance R_{yx} value of the TI/FeTe heterostructure is determined by the jointed contribution of the Hall resistance of the FeTe layer $R_{yx}{}^{FeTe}$ and the modified Hall resistance of the TI layer $R_{yx}{}^{TI} * \left(\frac{R_{xx}{}^{FeTe}}{R_{xx}{}^{TI}}\right)^2$. For all $(Bi_{1-x}Sb_x)_2Te_3$/FeTe heterostructures, both $R_{xx}{}^{FeTe}$ and $R_{yx}{}^{FeTe}$ are assumed to be constant. Systematic change in *x* can affect both $R_{xx}{}^{TI}$ and $R_{yx}{}^{TI}$. For $0 \leqslant x \leqslant 0.6$, the value of $\frac{R_{yx}{}^{TI}}{\left(R_{xx}{}^{TI}\right)^2}$ remains relatively insensitive to *x* and thus results in systematic behaviors in R_{yx} of the TI/FeTe heterostructures. However, for $0.7 \leqslant x \leqslant 1.0$,

specifically when the chemical potential is near the Dirac point of the TI layer, the value of $\frac{R_{yx}{}^{TI}}{\left(R_{xx}{}^{TI}\right)^2}$ becomes more sensitive to x and thus leads to fluctuations in the Hall traces. We note that the carrier type of the FeTe layer is n-type (Supplementary Figs. S16 and S17), a change in the Hall trace sign of the $(Bi_{1-x}Sb_x)_2Te_3$/FeTe heterostructures is in the range of $0.6 < x < 0.7$, as opposed to occurring beyond x=0.8, where the chemical potential crosses the Dirac point based on ARPES (Figs. 2 and 4a). This observation also suggests that there is no substantial charge transfer effect between the $(Bi_{1-x}Sb_x)_2Te_3$ layer and FeTe layers.

Supplementary References

1 Miao, L., Xu, Y. S., Zhang, W. H., Older, D., Breitweiser, S. A., Kotta, E., He, H. W., Suzuki, T., Denlinger, J. D., Biswas, R. R., Checkelsky, J. G., Wu, W. D. & Wray, L. A. Observation of a topological insulator Dirac cone reshaped by non-magnetic impurity resonance. *npj Quantum Mater.* **3**, 29 (2018).

2 Yokota, K. & Katayama, S. Thermal-Conductivities of $(Bi_{1-x}Sb_x)_2Te_3$ and $Bi_2(Te_{1-y}Se_y)_3$ Compounds. *Jpn. J. Appl. Phys.* **12**, 1205-1214 (1973).

3 Chang, C.-Z., Liu, C.-X. & MacDonald, A. H. Colloquium: Quantum anomalous Hall effect. *Rev. Mod. Phys.* **95**, 011002 (2023).

4 Zhang, Y., He, K., Chang, C. Z., Song, C. L., Wang, L. L., Chen, X., Jia, J. F., Fang, Z., Dai, X., Shan, W. Y., Shen, S. Q., Niu, Q., Qi, X. L., Zhang, S. C., Ma, X. C. & Xue, Q. K. Crossover of the Three-Dimensional Topological Insulator Bi_2Se_3 to the Two-Dimensional Limit. *Nat. Phys.* **6**, 584-588 (2010).

5 Li, Y. Y., Wang, G. A., Zhu, X. G., Liu, M. H., Ye, C., Chen, X., Wang, Y. Y., He, K., Wang, L. L., Ma, X. C., Zhang, H. J., Dai, X., Fang, Z., Xie, X. C., Liu, Y., Qi, X. L., Jia, J. F., Zhang, S. C. & Xue, Q. K. Intrinsic Topological Insulator Bi_2Te_3 Thin Films on Si and Their Thickness Limit. *Adv. Mater.* **22**, 4002-4007 (2010).

6 Jiang, Y. P., Wang, Y. L., Chen, M., Li, Z., Song, C. L., He, K., Wang, L. L., Chen, X., Ma, X. C. & Xue, Q. K. Landau Quantization and the Thickness Limit of Topological Insulator Thin Films of Sb_2Te_3. *Phys. Rev. Lett.* **108**, 016401 (2012).

7 Liu, C. X., Zhang, H., Yan, B. H., Qi, X. L., Frauenheim, T., Dai, X., Fang, Z. & Zhang, S. C. Oscillatory Crossover from Two-Dimensional to Three-Dimensional Topological Insulators. *Phys. Rev. B* **81**, 041307 (2010).

8 Lu, H. Z., Shan, W. Y., Yao, W., Niu, Q. & Shen, S. Q. Massive Dirac fermions and spin physics in an ultrathin film of topological insulator. *Phys. Rev. B* **81**, 115407 (2010).

9 Van Vleck, J. H. *The theory of electric and magnetic susceptibilities*. (Oxford University Press, 1965).

10 Bloembergen, N. & Rowland, T. J. Nuclear Spin Exchange in Solids - Tl^{203} and Tl^{205} Magnetic Resonance in Thallium and Thallic Oxide. *Phys. Rev.* **97**, 1679-1698 (1955).

11 Yu, R., Zhang, W., Zhang, H. J., Zhang, S. C., Dai, X. & Fang, Z. Quantized Anomalous Hall Effect in Magnetic Topological Insulators. *Science* **329**, 61-64 (2010).

12 Zhang, J. S., Chang, C. Z., Zhang, Z. C., Wen, J., Feng, X., Li, K., Liu, M. H., He, K., Wang, L. L., Chen, X., Xue, Q. K., Ma, X. C. & Wang, Y. Y. Band Structure Engineering in $(Bi_{1-x}Sb_x)_2Te_3$ Ternary Topological Insulators. *Nat. Commun.* **2**, 574 (2011).
